# Metformin for aging and cancer prevention

**DOI:** 10.18632/aging.100230

**Published:** 2010-11-11

**Authors:** Vladimir N. Anisimov

**Affiliations:** Department of Carcinogenesis and Oncogerontology, N.N. Petrov Research Institute of Oncology, St.Petersburg 197758, Russia

**Keywords:** metformin, biguanides, life span, aging, cancer prevention

## Abstract

Studies in mammals have led to the suggestion that hyperglycemia and hyperinsulinemia are important factors in aging. Insulin/insulin-like growth factor 1 (IGF-1) signaling molecules that have been linked to longevity include daf-2 and InR and their homologues in mammals, and inactivation of the corresponding genes increases life span in nematodes, fruit flies and mice. It is possible that the life-prolonging effect of caloric restriction is due to decreasing IGF-1 levels. Evidence has emerged that antidiabetic drugs are promising candidates for both life span extension and prevention of cancer. Thus, antidiabetic drugs postpone spontaneous carcinogenesis in mice and rats, as well as chemical and radiation carcinogenesis in mice, rats and hamsters. Furthermore metformin seems to decrease cancer risk in diabetic patients.

## INTRODUCTION

The effects of factors or drugs that increase life span (geroprotectors) on spontaneous tumor development may provide important clues to the interactions of aging and carcinogenesis. A number of substances were suggested as life span extension means [1-3]. Being suggested on the current knowledge on factors and mechanisms or theories of aging these pharmacological interventions in the aging process sometime were followed some unfavorable effects. The comparison of the data on the mechanisms of action of geroprotectors with its influence on the development of spontaneous and experimentally induced tumors permits to deepen our understanding of interactions between two fundamental biological processes - aging and carcinogenesis [1,2,4]. The main goal of this review is critical evaluation of available data on effects of antidiabetic drugs on aging development in experimental animals and perspectives of practical use of the drugs for cancer prevention and enhance of healthy aging in humans.

Calorie restriction (CR) is the only known intervention in mammals that has been shown consistently to increase life span, reduce incidence and retard the onset of age-related diseases, including cancer and diabetes. CR has also been shown to increase the resistance to stress and toxicity, and maintain function and vitality in laboratory mammals of younger ages [3,5-7]. Studies in CR rhesus monkeys have produced physiological responses strikingly similar to those observed in rodents [3,8,9]. Emerging data from these studies suggest that long-term CR will reduce morbidity and mortality in primates, and thus may exert beneficial “anti-aging” effects in humans [3,8-11]. It is worthy to note that alongside with point of view that CR can work for human beings, there are authors who believe that to early to decide it or CR cannot work for human beings [3,12-19].

The crucial event of the action of CR is the reduction in the levels of insulin and insulin-like growth factor-1 (IGF-1) and also increases insulin sensitivity in rodents [5,12] as well as in monkeys [9]. In *C. elegans* and *D.melanogaster*, the mutation modification of genes operating in the signal transduction from insulin receptor to transcription factor *daf-16* (*age-1, daf-2, InR*, etc.) are strongly associated with longevity [10,19,20]. Whole-genome analysis of gene expression during aging of nematode worm *C. elegans* provided new evidence on the role of insulin homologue genes and SIR2 homologues in longevity by interacting with the *daf-2/age-1* insulin-like signaling pathway and regulating downstream targets [21].

Activity of *daf-16* counteracts both aging and tumor growth in *C. elegans*. These two processes are linked in nature, because tumor rates invariably rise as an animal ages. Pinkston-Gosse and Kenyon [22] have identified 29 *daf-16*/FOXO-regulated genes that act in the insulin/IGF-1 pathway to influence *C. elegans* tumor growth. Some of these genes are required for the entire effect that *daf-2* mutations have on cell death, but none appear to be required for the entire effect on cell division. Because both increased apoptosis and reduced cell proliferation contribute to the tumor-protective effects of *daf-2* mutations [23], together there downstream genes are likely to act in a cumulative fashion to influence tumor growth, much as downstream targets of *daf-16* appear to act cumulatively to influence lifespan [24]. It was shown that almost half of the genes that affect tumor growth also affect the lifespan of animals that do not have tumors, indicating that the ability of the insulin/IGF-1 pathway to couple longevity and tumor resistance extends downstream of DAF-16 [21].

*Daf-2* and *InR* are structural homologues of tyrosine kinase receptor in vertebrata that includes the insulin receptor and the IGF-1 receptor. It was shown that in vertebrata the insulin receptor regulates energy metabolism whereas IGF-1 receptor promotes a growth. During last years series of elegant experiments in mice and rats in which this or that key elements of the insulin/IGF-1 signaling pathway were genetically modified provide an evidence of the involvement of the system in the control of mammalian aging and longevity [25]. Recently it was shown that in mice, less insulin receptor substrate-2 (Irs2) signaling throughout the body or just in the brain extended life span up to 18% [26]. Kapeller et al. [27] showed that partial inactivation of brain IGF receptors (IGF-1R) in embryonic brain selectively inhibited GH and IGF-1 pathways after birth. This causes growth retardation, smaller adult size, and metabolic alterations, and led to delayed mortality and longer mean life span. The mean life span in bIGF1RKO^+/-^ mice was increased by 9.3% (p<0.05) as compared with control mice, and tumor incidence was 44% and 53%, correspondingly (p>0.05).

Selman et al. [28] measured the life span of mice lacking either insulin receptor substrate (IRS) 1 or 2, the major intracellular effectors of the insulin/IGF-1 signaling (IIS) receptors. It was observed that female *Irs1^-/-^* mice are long-lived. Furthermore, they displayed resistance to a range of age-sensitive markers of aging including skin, bone, immune, and motor disfunction. These improvement in health were seen despite mild, lifelong insulin resistance. Thus, enhanced insulin sensitivity is not a prerequisite for IIS. *Irs1^-/-^*female mice also displayed normal anterior pituitary function, distinguishing them from long-living somatotrophic axis mutants. In contrast, Irs2*^-/-^* mice were short-lived, whereas *Irs1^+/-^* and *Irs2^+/-^* mice of both sexes showed normal life spans.

It was shown that the incidence of mutations in insulin regulatory region (IRE) of APO C-III T-455 C directly correlates with longevity in humans. This is the first evidence showing that mutation located downstream to *daf-16* in insulin signal transduction system is associated with longevity [29]. It is worth noting that centenarians display lower degree of resistance to insulin and lower degree of oxidative stress as compared with elderly persons before 90 years [30,31]. The authors suggest that centenarians may have been selected for appropriate insulin regulation as well as for the appropriate regulation of tyrosine hydroxylase (TH) gene, whose product the rate is limiting in the synthesis of catecholamines, stress-response mediators. It was shown that catecholamine may increase free radical production through induction of the metabolic rate and auto oxidation in diabetic animals [32]. A study on aging parameters of young (up to 39) and old (over 70) individuals having similar IGF-1 serum levels provides evidence of important role of this peptide for life potential [33]. Roth et al. [34] analyzed data from the Baltimore Longitudinal Study of Aging and reported that survival was greater in men who maintained lower insulin level. In women, genetic variation causing reduced insulin/IGF-1 signaling pathway activation is beneficial for old age survival [35].

Hyperglycemia is an important aging factor involved in generation of advanced glycosylation end products (AGEs) [36-38]. Untreated diabetics with elevated glucose levels suffer many manifestations of accelerated aging, such as impaired wound healing, cataracts, vascular and microvascular damage [39]. The accumulation of the AGE, pentosidine, is accelerated in diabetics and has been suggested to be a reliable biomarker of aging [36]. The action of insulin provides the major modulator of glucose storage and utilization. It is important to stress that hyperinsulinemia is also an important factor in development of cancer [39,40-45].

The concept of CR mimetics is now being intensively explored [11,46-51]. CR mimetics involves inter-ventions that produce physiological and anti-aging effects similar to CR. Reviewing the available data on the benefits and adverse effects of CR and genetic modifications, Longo and Finch [52] suggested three categories of drugs which may have potential to prevent or postpone age-related diseases and extend life span: drugs that (i) stimulate dwarf mutations and therefore decrease pituitary production of growth hormone (GH); (ii) prevent IGF-1 release from the liver, or (iii) decrease IGF-1 signaling by the action on either extracellular or intracellular targets.

The antidiabetic drugs, phenformin (1-phenylethylbiguanide), buformin (1-butylbiguanide hydrochloride) and metformin (N,N-dimethyl-biguanide) were observed to reduce hyperglycemia, improve glucose utilization, reduce free fatty acid utilization, gluconeogenesis, serum lipids, insulin, IGF-1, reduce body weight and decrease metabolic immunodepression both in humans and rodents [39,40,53,54]. Nowadays, phenformin is not used in clinical practice due to it side effects (mainly lactic acidosis) observed in patients with non-compensated diabetes. It is worthy of note that during more than 10-year-long experience of administration of phenformin to patients without advanced diabetes Vladimir M. Dilman and Lev M.Berstein [55-61] observed no cases of lactic acidosis or any other side effects. We believe that the analysis of results of long-term administration of this drug as well as another antidiabetic biguanides (buformin and metformin) to non-diabetic animals is seems very important for understanding of links between insulin and longevity on the one hand and between insulin and cancer on the other hand.

### Biguanides and Life Span

Fourty years ago, it was suggested to use biguanides as a potential anti-aging treatment [39,62]. There are data on effect of the drugs on life span in worms, mice and rats. Buformin was supplemented to nutrient medium in various concentrations (from 1.0 to 0.00001 mg/ml) during the larvae stage and over the life span of *C.elegans.* The drug given at the concentration of 0.1 mg/ml increased the mean life span of the worms by 23.4% (p < 0.05) and the maximum life span by 26.1% as compared to the controls [63]. Metformin supplementation (50 mM dose) was shown to increase the mean life span, but not maximum, of *C. elegans*, although 10 or 100 mM doses showed no significant life span benefit [64]. The authors have shown that metformin prolongs nematode healthspan, slowing lipofuscin accumulation, extending mean life span, and prolonging youthful locomotor ability in a dose-dependent manner. Genetic data suggest the metformin acts through a mechanism similar to that operative in eating-impaired CR mutants, but independent of insulin signaling pathway. Energy sensor AMPK and AMPK-activating kinase LKB1, which are activated in mammals by metformin treatment [65,66], are essential for health benefits in *C. elegans*, suggesting that metformin engages a metabolic loop conserved across phyla [64]. It was also shown metformin activated SKN-1/Nrf2, oxidative stress-responsive transcription factor.

The available data on effect of antidiabetic biguanides on life span in mice and rats are summarized in the Table 1. Female C3H/Sn mice were kept from the age of 3.5 months at standard *ad libitum* diet were given phenformin 5 times a week orally at a single dose 2 mg/mouse until a natural death [67]. The treatment with phenformin prolonged the mean life span of mice by 21% (p < 0.05), the mean life span of last 10% survivors by 28% and the maximum life span by 5.5 months (by 26%) in comparison with the control. At the time of death of the last mice in the control group 42% of phenformin-treated mice were alive. In this study food consumption in control and drug-exposed groups was not measured.

Phenformin was given 5 times a week to female outbred LIO rats starting from the age of 3.5 month until a natural death in a single dose of 5 mg/rat/day orally [1,73]. Administration of phenformin failed to influence the mean life span in rats. At the same time, the mean life span of the last 10% survivors was increased by 10% (p < 0.005), and maximum life span was increased by 3 months (+ 10%) in comparison with the controls. The treatment with phenformin slightly decreased the body weight of rats in comparison with the control (p > 0.05). The disturbances in the estrus function observed in 36 % of 15-16-month old rats of the control group and only in 7% of rats in phenformin-treated group (p < 0.05).

Buformin was given 5 times a week to female LIO rats starting from the age of 3.5 month until a natural death in a single dose of 5 mg/rat/day orally [73,74]. The treatment slightly increased mean life span of rats (by 7%; p > 0.05). The mean life span of the last 10% survivors increased by 12% (p < 0.05) and the maximum life span increased by 2 months (+5.5%) as compared with controls. The body weight of rats treated with buformin was slightly (5.2 to 9.4%) but statistically significantly (p < 0.05) decreased in comparison with the control from the age of 12 months to 20 months (p < 0.05). At the age of 16-18 months 38% of control rats revealed the disturbances in the estrus cycle persistent estrus, repetitive pseudo-pregnancies or anestrous), whereas in females treated with buformin these disturbances were observed only in 9% of rats (p < 0.05). Again, in both these studies food consumption in control and drug-exposed groups was not measured.

Long-term administration of metformin (100 mg/kg in drinking water) slightly decreased the food consumption but did not changed the body weight or temperature, slowed down the age-related rise in blood glucose and triglycerides level, as well as the age-related switch-off of estrous function, prolonged the mean life span by 8% (p < 0.05), the mean life span of last 10% survivors by 13.1%, and the maximum life span by 1 month in transgenic HER-2/neu mice in comparison with the control animals [68]. The demographic aging rate represented by the estimate of respective Gompertz's parameter was decreased 2.26 times. The reduction of the serum level of cholesterol and beta-lipoproteins was observed in metformin-treated transgenic HER-2/neu female mice [68]. Metformin treatment decreased the food consumption in these mice at the age of 4 and 6 months, suggesting that “voluntary CR” may be the sourse of the effects of metformin [3]. In our new experiment metformin was given to male and female 129/Sv mice [72]. It was observed slight increase in mean and maximum life span in females but reduction of the mean life span by 13.4% in males.

**Table 1.** Effect of antidiabetic drugs on life span in mice and rats

Strain	Sex	Treatment	No. of animals	Life span, days			References
				Mean	Last 10% of survivors	Maximum	
**Mouse**							
C3H/Sn	Female	Control	30	450 ± 23.4	631 ± 11.4	643	[67]
		Phenformin	24	545 ± 39.2 (+21.1%)	810 ± 0 * (+28.4%)	810 (+26%)	
FVB/N	Female	Control	34	264 ± 3.5	297 ± 7.3	311	[68]
		Metformin	32	285 ± 5.2 (+8.0%)	336 ± 2.7 (+13.1%)*	340 (+16.2%)	
FVB/N	Female	Control	15	285 ± 12	396 ± 0	396	[69]
		Metformin	20	304 ± 10	352 ± 7	359	
SHR	Female	Control	50	388 ± 29.2	727 ± 22.5	814	[70]
		Metformin	50	535 ± 31.9* (+37.9%)	878 ± 6.6* (+20.8%)	898 (+10.3%)	
NMRI	Female	Control	50	346 ± 11.9	480 ± 9.2	511	[71]
		Diabenol	50	369 ± 12.9	504 ± 6.4* (+5.9%)	518	
129/Sv	Male	Control	41	662 ± 27.7	951 ± 32.3	1029	[72]
		Metformin	46	573 ± 26.5 (-13.4%)*	931 ± 30.4	1044	
129/Sv	Female	Control	47	706 ± 20.8	910 ± 8.9	930	
		Metformin	48	742 ± 16.3 (+5.1%)	913 ± 19.2	966 (+3.9%)	
**Rat**							
LIO	Female	Control	41	652 ± 27.3	885 ± 11.3	919	[1,73]
		Phenformin	44	652 ± 28.7	974 ± 16.2** (+10.1%)	1009 (+9.8%)	
	Female	Control	74	687 ± 19.2	925 ± 22.5	1054	[74]
		Buformin	42	737 ± 26.4 (+7.3%)	1036± 38.9* (+12%)	1112 (+5.5%)	
Fischer-344	Male	Control	31	796 ± 170	1039 ± 29.6	1065	[75]
		Metformin	40	815 ± 186	1061 ± 2.5	1062	

The difference with control is significant: * - p < 0.05 ; ** p < 0.01 (Student's test)

The chronic treatment of female outbreed SHR mice with metformin (100 mg/kg in drinking water) slightly modified the food consumption but decreased the body weight after the age of 2 months (p<0.01), increased the mean life span of last 10% survivors by 20.8% (p<0.01), and maximum life span by 2.8 months (+10.3%) in comparison with the control SHR mice [700]. The treatment with metformin failed to influence blood estradiol concentration and spontaneous tumor incidence in female SHR mice.

Transgenic mice with Hungtington's disease (HD) (the R6/2 line expressing exon 1 of the hungtington protein including ~130 glutamine repeats) were given metformin in drinking water (2 or 5 mg/ml) starting from the age of 5 weeks [76]. Metformin treatment significantly prolonged (by 20.1%) the survival time of male (but not female) HD mice at the 2 mg/ml dose (~ 300 mg/kg/day) without affecting fasting blood glucose level. This dose of the drug also decreased hind limb clasping time in 11-week-old mice. The higher dose of metfromin did not prolong life span, and neither dose was effective in female HD mice.

In the study of Smith et al. [75], six month old male F344 rats were randomized to one of four diet: control, calorie restricted (CR), metformin (300 mg/kg/day) and pair fed to metformin. The CR group had significantly reduced food intake and body weight throughout the study. Body weight was significantly reduced in the metformin group compared with control during the middle of the study, despite similar weekly food intake. There were no significant differences in the mean life span or the mean of the last surviving 10% of each group in the CR, metformin and pair fed groups compared with control. However, the aging rate estimate (α - slope, rate of increase of mortality) of the Gompertz model of the control group alone was significantly different from the three other groups, reflecting the early deaths in the CR, metformin and pair fed groups. CR significantly increased life span in the 25th quantile but not the 50th, 75th, or 90th quantile. The groups of rats exposed to metformin or to the pair feeding were not significantly different from controls at any quantile [75]. The authors stressed the one limitation of this study - the lack of a robust CR response for extension of maximum life span which has been observed in another the CR study using the same strain of rats [19]. The reduced efficacy of CR in this study might provide a partial explanation for the lack of a significant increase with metformin treatment. In addition to the dampened CR response, metformin treatment did not significantly affect glucose/insulin levels in this study. The metformin concentration utilized in the diet is approximately 10 times that of the highest dose used in human treatments, implying that any increase necessary to observe life span benefits is questionable for a human application [75].

There are only few data on the effect of other than biguanides antidiabetic drugs on life span of animals. The effects of new antidiabetic drug Diabenol® (9-β-diethyl-aminoethyl-2,3-dihydro-imidazo-(1, 2-α) benzi-midazol dihydrochloride) on life span of NMRI and transgenic HER-2/neu mice have been studied [71]. Diabenol was synthesized in Rostov State University and its hypoglycemic activity was evaluated as 1.5 times more effective than of maninil (glibenclamide) and equal to the effect of glyclazide (pioglitazone) in rats, rabbits and dogs [77,78]. Diabenol restores increases tissue susceptibility to insulin and extends hypoglycemic effect of the hormone. It increases glucose utilization in glucose loading test in the old obese rats. It was suggested that diabenol influence insulin receptors in peripheral tissues. Diabenol increases uptake of glucose by isolated rat diaphragm *in vitro* both without supplementation of insulin into the medium or with supplemented insulin. Diabenol also decreases platelet and erythrocyte aggregation and blood viscosity, inhibits mutagenic effect of 2-acetylaminofluorene and has antioxidant activity [77,79,80]. It is shown that treatment with the drug failed influence body weight gain dynamics, food and water consumption and the body temperature, slowed down age-related disturbances in estrous function and increased life span of all and 10% most long-living NMRI mice [71]. Diabenol treatment slowed down age-related changes in estrous function in HER-2/neu mice, failed influence survival of these mice.

Thus, available data gives evidence that antidiabetic drugs in some cases can increase survival of rodents (Table 1). This effect was not observed in all experiments and varied depending on strain and species of animals. Female mice and rats have been treated in the majority of these studies. The experiments with males of different strains will be useful for conclusion on geroprotective potential of antidiabetic biguanides.

Metformin is now a widely prescribed medication for treating type 2 diabetes. In addition to improving the metabolic profile of diabetes, increased survival from all-cause mortality has been associated with metformin treatment in both diabetic and cardiovascular disease patients [81,82].

### Biguanides and Endocrine System

Parameters of reproductive function are among most valuable biomarkers of aging [83]. The treatment with phenformin decreased hypothalamic threshold of the sensitivity to feedback inhibition by estrogens [84,85], which is one of the most important mechanisms regulating age-related decline and switch-off of the reproductive function [39,62,86,87]. It is worthy of note that another antidiabetic biguanide, metformin, may improve menstrual regularity, leading to spontaneous ovulation, and enhance the induction of ovulation with clomiphene citrate in women with polycystic ovary syndrome [88,89]. The treatment with phenformin also decreased hypothalamic threshold sensitivity to feedback regulation by glucocorticoids and by metabolic stimuli (glucose and insulin) [39]. It was recently shown that elements involved in the insulin/IGF-1 signaling pathway are regulated at the expression and/or functional level in the central nervous system. This regulation may play a role in the brain's insulin resistance [90], in the control of ovarian follicular development and ovulation [91], and brain's control of life span [92,93]. Antidiabetic biguanides also alleviated age-related metabolic immunodepression [39,40]. These mechanisms can be involved in geroprotective effect of biguanides.

Treatment with chromium picolinate which elevated the insulin sensitivity in several tissues, including hypothalamus, significantly increased the mean life span and decreased the development of age-related pathology in rats [94]. It was hypothesized that antidiabetic biguanides and possibly chromium picolinate, have regulate tyrosine hydroxylase and insulin/IGF-1 signaling pathway genes both associated with longevity [10,95,96]. It was shown that the polymorphism at TH-INS locus affects non-insulin dependent type 2 diabetes [97], and is associated with hypothalamic obesity [98], polycystic ovary syndrome [99], hypertriglyceridemia and atherosclerosis [100].

### Anti-tumor Effects of Biguanides

Nowadays there are a burst of data on anti-tumor effects of biguanides both *in vitro* and *in vivo* [for review see: 45,69,101,102]. In this paper we shall not to discuss this aspect of the biguanides' potential. However the capacity of the biguanides to prevent spontaneous and tumorigenesis induced by chemical carcinogens or ionizing irradiation will be briefly analyzed. Long-term treatment with phenformin significantly inhibited (by 4.0-fold, p < 0.01) the incidence of spontaneous mammary adenocarcinomas in female C3H/Sn mice [67]. The tumor yield curve rise was also significantly slowed down as a result of the treatment. The treatment with phenformin was followed by 1.6-fold decrease in total spontaneous tumor incidence in rats, whereas total tumor incidence was decreased by 49.5% in buformin-treated rats [73,74].

The anticarcinogenic effect of antidiabetic biguanides has been demonstrated in several models of induced carcinogenesis (Table 2).

Daily oral administration of phenformin or buformin suppressed 7,12-dimethylbenz(a)anthracene (DMBA)-induced mammary tumor development in rats [73,111,112]. Phenformin-treated rats revealed a tendency toward a decrease in serum insulin level. The treatment with phenformin normalised the tolerance to glucose and serum insulin and IGF-1 level in rats exposed to intravenous injections of N-nitro-somethylurea (NMU) and inhibited mammary carcinogenesis in these animals [113]. Treatment of rats with 1,2-dimethylhydrazine (DMH) (once a week during 4 weeks) caused the decrease in the level of biogenic amines, particularly, of dopamine in the hypothalamus, the decrease of glucose tolerance and the increase of the blood level of insulin and triglycerides. The exposure to DMH also caused the inhibition of lymphocyte blastogenic response to phytohemagglutinin and lipopolysaccharide, the decrease in the level of antibody produced against sheep erythrocytes and the decrease in phagocytic activity of macrophages [117]. Administration of phenformin started from the 1st injection of the carcinogen restored all the above mentioned immunological indices and inhibited DMH-induced colon carcinogenesis [117,118]. It is worthy to note that colon 38 adenocarcinoma growth was significantly inhibited in liver-specific IGF-1-deficient mice whereas injections with recombinant human IGF-1 displayed sufficiently promoted the tumour growth and metastasing [122].

A decrease of glucose utilisation in the oral glucose tolerance test was found in the 3-month-old female progeny of rats exposed to NMU on the 21st day of pregnancy [115]. The serum insulin level was not differ from the control, but the cholesterol level was higher in offspring of NMU-treated rats as compared with the control. Postnatal treatment with buformin started from the age of 2 months significantly inhibited the development of malignant neurogenic tumors in rats transplancentally exposed to NMU. Similar results have been observed in rats exposed transplacentally to N-nitrosoethylurea (NEU) and postnatally to phenformin [116]. Authors observed the decrease of development of nervous system and renal tumors induced transplacentally with NEU. The treatment with phenformin inhibited also the carcinogenesis induced by a single total-body X-rays irradiation in rats [119].

**Table 2.** Effect of antidiabetic drugs on carcinogenesis in rodents

Drug	Carcinogen	Main target(s)	Effect	References
***Mouse***				
Phenformin	Spontaneous	Mammary gland	Inhibition	[67]
Phenformin	20-methylcholantrene	Subcutaneos soft tissues	Inhibition	[103]
Diabenol	HER2/neu	Mammary gland	Inhibition	[71]
Metformin	HER2/neu	Mammary gland	Inhibition	[68, 69]
Metformin	Spontaneous	Mammary gland	No effects	[70]
Diabenol	Spontaneous	Mammary gland	Inhibition	[71]
Metformin	Spontaneous, Apc^Min/+^	Small intestines	Inhibition	[104]
Metformin	Spontaneous, PTEN+/-	Lymphoma, intestine	Inhibition	[105]
Phenformin	Spontaneous, PTEN+/-		Inhibition	
Metformin	Azoxymethane	Colon	Inhibition	[106]
Metformin	Estradiol, tamoxifen	Endometrium	Inhibition	[107]
Metformin	Benzo(a)pyrene	Cervix utery and vagina	Inhibition	[108]
Metformin	Benzo(a)pyrene	Skin	Inhibition	[109]
Metformin	NNK	Lung	Inhibition	[110]
***Rat***				
Buformin	Spontaneous	Total incidence	Inhibition	[73,74]
Phenformin	Spontaneous	Total incidence	Inhibition	[73]
Phenformin	DMBA	Mammary gland	Inhibition	[111,112]
Phenformin	N-nitrosomethylurea	Mammary gland	Inhibition	[113]
Buformin	DMBA	Mammary gland	Inhibition	[114]
Buformin	N-nitrosomethylurea, transplacentally	Nervous system	Inhibition	[115]
Phenformin	N-nitrosoethylurea, transplacentally	Nervous system, kidney	Inhibition	[116]
Phenformin	1,2-dimethylhydrazine	Colon	Inhibition	[117,118]
Diabenol	1,2-dimethylhydrazine	Colon	Inhibition	[71]
Phenformin	X-rays	Total incidence	Inhibition	[119]
Metfromin	N-nitrosomethylurea	Mammary gland	Inhibition	[120]
***Hamster***				
Metformin	N-nitrosobis(2-oxoprop-yl)amine	Pancreas	Inhibition	[121]

Abbreviations: DMH - 1,2-dimethylhydrazine; MCA - 20-methylcholanthrene; NBOPA - N-nitrosobis-(2-oxopropyl)amine; NEU - N-nitrosoethylurea; NMU - N-nitrosomethylurea; NNK - 4-(methylnitrosamino)-1-(3-pyridyl)-1-butanone; X-rays - total-body X-ray irradiation.

Vinnitski and Iakumenko [103] have shown that treatment with phenformin increased the immunological reactivity and inhibited carcinogenesis induced by s.c. administration of 20-methyl-cholanthrene in BALB/c mice.

It was shown that metformin suppresses both azoxymethane-induced colorectcal aberrant crypt foci [106] and lung carcinogenesis induced by tobacco carcinogen in mice [110]. It inhibits also benzo(a)pyrene-induced skin and cervico-vaginal carcinogenesis in mice [108,109].

In high fat-fed hamsters, the treatment with N-nitrosobis-(2-oxopropyl) amine was followed by the development of pancreatic malignancies in 50% of cases, whereas no tumors were found in the hamsters treated with the carcinogen and metformin [121].

The effects of new antidiabetic drug Diabenol® on spontaneous tumor incidence in NMRI and transgenic HER-2/neu mice as well as on colon carcinogenesis induced by 1,2-dimethylhydrazine in rats are studied [71]. The treatment with diabenol inhibited spon-taneous tumor incidence and increased the mammary tumor latency in these mice. Diabenol treatment slowed down age-related changes in estrous function in HER-2/neu mice, failed influence survival of these mice and slightly inhibited the incidence and decreased the size of mammary adenocarcinoma metastases into the lung. In rats exposed to DMH, treatment with diabenol significantly inhibited multiplicity of all colon tumors, decreased by 2.2 times the incidence of carcinomas in ascending colon and by 3.1 times their multiplicity. Treatment with diabenol was followed by higher incidence of exophytic and well-differentiated colon tumors as compared with the control rats exposed to the carcinogen alone (76.3% and 50%, and 47.4% and 14.7%, respectively). Thus, antidiabetic drugs inhibits spontaneous and induced carcinogenesis in rodents.

### Antidiabetic Drugs and Cancer Risk in Human

There are evidences that insulin resistance or some other aspect of type 2 diabetes may promote breast and some other cancers [39,123,124]. Biguanides were used as a component of the so-called metabolic rehabilitation of breast and colon cancer patients [39,55,56]. The total of 324 patients (182 with breast cancer and 142 with colon cancer) treated by surgery of the primary tumor were andomly divided into control and treatment groups. In the latter group, diet with reduction of saturated fats and cholesterol was complemented by biguanides or hypolypidemic drugs (mainly clofibrate in breast cancer patients). Among patients treated with biguanides 304 were given the drug more than 3 years and 15% more than 5 years. The authors reported an overall improvement in the cumulative survival by 3-6 and 4-7 years of observation in groups with breast and colon cancer, respectively, as well as a slight decrease in frequency of primary multiple neoplasms and metachronous tumors in the contralateral breast [39,55-61]. Evans et al. [125] reported results of a pilot case-control study on 11 876 diabetic patients treated or not treated with metformin. Authors collated information about use of metformin for all cases and controls and calculated unadjusted odds ratios of cancer risk using conditional logistic regression. More than a third (336; 36.4%) of the cases had been given at least on prescription of metformin in the year before their index date compared with 732 (39.7%) of the controls. The unadjusted odds ratio of cancer risk was estimated as 0.86 (95% confidential interval - 0.73 to 1.02). The unadjusted odds ratio for any exposure to the drug since 1993 was 0.79 (0.67 to 0.93). It is worthy to note that the authors suggested a dose-response relationship: adjusted odds ratio for patients dispensed total amount 14 - 672 g of metformin was 0.83 (0.65 to 1.06), dispensed 673 - 964 g of metformin - 0.86 (0.68 to 1.10) and dispensed more than 964 g - 0.57 (0.43 to 0.75).

In another study, cancer-related mortality was compared among inception cohorts of metformin users and sulfonylurea monotherapy users [126]. There were 10,309 new users of metformin or sulfonylurea with an average follow-up of 5.4 ± 1.9 years. Cancer mortality over follow-up was 4.9% (162 of 3,340) for sulfonylurea monotherapy users, and 3.5 (245 of 6,969) for metformin users (p = 0.01 for χ2 test) and 5.8 (84 of 1,443) for patients who used insulin. Multivariate adjustment has shown that the sulfonylurea cohort had greater cancer-related mortality compared with the metformin cohort (1.3 (95% CI 1.1-1.6); p = 0.012), whereas insulin use was associated with an adjusted cancer-related mortality of 1.9 (1.5 - 2.4; p < 0.0001). In a prospective study, it was found (after a median follow-up time of 9.6 years) that metformin use at baseline was associated with less cancer-related mortality [127]. 1 353 patients with type 2 diabetes were enrolled in the project. Of the patients, 570 died, of which 122 died of malignancies. The standardized mortality rations (SMRs) for cancer mortality was 1.46 (95% CI 1.22 - 1.76). In patients taking metformin compared with patients not taking metformin at baseline, the adjusted hazard ration for cancer mortality was 0.43 (95% CI 0.23 - 0.80). Cochrane report stressed that there were either insufficient or no data on the relative efficacy of metformin for preventing the development of diabetes, cardiovascular disease, or endometrial cancer [128].

### Mechanisms of Effects of Biguanides on Aging and Cancer

It is worthy to note that studies of metfromin distribution in mice showed that 2 hour after a single oral dose of 14C metfromin (150 mg/kg) there was a measurable accumulation of the drug in brain tissue [129]. It was shown that orally administered metformin increases brain AMPK activation [76]. This suggests that metfromin crossed the blood-brain barrier and exert a pharmacological effect in intact brain.

Data on molecular mechanisms of inhibitory effect of biguanides on tumor growth have been discussed in several recent papers [49-51,101,102,130-137]. The formation and accumulation of advanced glycation end products (AGE) in various tissues are known to be involved in aging process and complications of long-term diabetes [36,37]. Effects of biguanides buformin and metformin on AGE formation have been studied *in vitro* [138]. It was shown that both drugs are potent inhibitors of AGE formation. In a descriptive clinical trial, 6-month-long metformin treatment of women with polycystic ovary syndrome (PCOS) significantly reduced AGE levels [139]. The AGE formation contributes in the increase atherogenic oxidative modification of low-density lipoproteins (LDL). The impact of glycemic control on the parameters of free radical oxidation has been studied in type 2 diabetic patients treated with metformin or sulfonylurea preparations [140]. Metformin was more effective in a decrease of glycated hemoglobin (Hb A1c), lipid peroxidation, malonic dialdehyde levels in the serum, and in increase of SOD and glutathione peroxidase in erythrocytes than sulfonylurea drugs. There are evidence that metformin primary inhibited complex I of the mitochondrial respiratory chain [141-143]. In mice, metformin treatment protected against adriamycin-induced increase in the serum level of malonic dialdehyde and decrease in the serum level of glutathione [144].

A number of reports concerning the cellular response to and molecular function of metformin have been published. Metformin has been shown to activate AMP-activated protein kinase [65] and inhibit the tyrosine phosphates activity [145]. Metformin has been reported to influence hepatic gene expression in cultured hepatocytes, with noted alteration in the expression of glucose-6-phosphatase (*G6pc*), gluco-kinase, and 3-hydroxy-3-methylglutaryl-Coenzyme A synthase 2 (*Hmgcs2*). These genes, which are related to glycolysis-gliconeogenesis or cholesterol metabolisms, were assumed to be critical for the action of metformin [146]. Global gene expression analysis has been performed in liver of obese diabetic *db/db* mice 3 hours after a single administration of metformin (400 mg/kg) [147]. It was identified 14 genes that showed at least a 1.5-fold difference in expression following metformin treatment, including a reduction of glucose-6-phospatase expression. It was recently observed that the antidiabetic biguanides metformin ans phenformin inhibit mTORC1 signaling, not only in the absence of TSC1/2 but also in the absence of AMPK, instead depended on the Rag GTPases [148].

Spindler [149] has found that 8 weeks of metformin treatment was superior to 8 weeks of caloric restriction at reproducing the specific changes in transcript levels produced by long-term caloric restriction in the liver of mice. The gene expression changes common to metformin and CR were associated with xenobiotic metabolism, cellular stress, energy metabolism, biosynthesis, signal transduction, and the cytoskeleton [150]. It was suggested that antidiabetic biguanides inhibit metabolic immunodepression, developed in animals exposed to carcinogenic agents which is similar to the immunodepression inherent to normal aging and specific age-related pathology [39,40]. If immunodepression is one of the important factors in carcinogenesis, then the elimination of metabolic immunodepression, which arises in the course of normal aging or under the influence of chemical carcinogens or ionizing radiation, can provide an anticarcinogenic prophylactic effect [1,39,40,117].

Although it is known that free radicals are produced during metabolic reactions, it is largely unknown which factor(s), of physiological or pathophysiological significance, modulate their production *in vivo*. It has been suggested that hyperinsulinemia may have increase free radicals and therefore promote aging, independent of glycemia [39,77,78,151,152]. Plasma levels of lipid hydroperoxides are higher, and antioxidant factors are lower in individuals who are resistant to insulin-stimulated glucose disposal but otherwise glucose tolerant, nonobese, and normotensive [77]. This finding indicates that enhanced oxidative stress is present before diabetes ensues and therefore cannot simply be explained by overt hyperglycemia. There is substantial evidence supporting the hypothesis that selective resistance to insulin-stimulated (muscle) glucose disposal and the consequential compensatory hyperinsulinemia trigger a variety of metabolic effects, likely resulting in accelerated oxidative stress and aging [39,77].

The antidiabetic biguanides inhibit fatty acid oxidation, gluconeogenesis in the liver, increase the availability of insulin receptors, decrease monoamine oxidase activity [53,54], increase sensitivity of hypothalamo-pituitary complex to negative feedback inhibition, and reduce excretion of glucocorticoid metabolites and dehydroepiandrosterone-sulfate [39]. These drugs have been proposed for the prevention of the age-related increase of cancer and atherosclerosis, and for retardation of the aging process [39,62]. It has been shown that administration of antidiabetic biguanides to patients with hyperlipidemia lowers the level of blood cholesterol, triglycerides, and b-lipoproteins. Biguanides also inhibits the development of atherosclerosis, reduces hyperinsulinemia in men with coronary artery disease. Its increases hypothalamo-pituitary sensitivity to inhibition by dexa-methasone and estrogens, causes restoration of estrous cycle in persistent-estrous old rats, improves cellular immunity in atherosclerotic and cancer patients, lowers blood IGF-1 levels in cancer and atherosclerotic patients with type IIb hyperlipoproteinemia. Recently it was shown that metformin decreases platelet superoxide anion production in diabetic patients [153]. There are observation that diabenol has similar effects [71]. Metformin increased glutathione (GSH) levels and diminished lipid peroxidation of T cells in mice thus the decrease of oxidative stress inhibited proliferation of T cells [154].

Morales et al. [155] showed that metformin ameliorate gentamicin-induced kidney injury through a mitochondria-dependent pathway, normalizing oxidative stress and restoring mitochondrial functional integrity [156]. It was shown that a single oral administration of metfromin (100, 500 or 2500 mg/kg) or multiple (4 or 8 weeks) treatment (100 or 500 mg/kg) was not toxic or cytotoxic in streptozotocin-induced diabetic or control non-diabetic rats. Moreover, metfromin protected rat bone marrow and sperm cells against the diabetes-induced genomic instability and decline in the cell proliferation [157]. Pretreatment with metformin reduced the doxorubicin-induced high frequency of micronuclei and protected against the doxorubicin-induced alteration of MDA and GSH in mice [144].

The available data on gender-specificity of the life span extension effect of metformin will require further studies. It is worthy to note that metformin is used clinically to treat hyperandrogenemia in females with polycystic ovary syndrome. Insulin resistance in these patients contributes to the increased levels of free androgen, and metformin, as an insulin sensitizing agent, is an effective treatment [158].

It was shown in mice deletion of ribosomal S6 protein kinase 1 (*S6K1*), a component of the nutrient-responsive mTOR (mammalian target of rapamycin) signaling pathway, led to increased life span and resistance to age-related pathologies, such as bone, immune, and motor dysfunction and loss of insulin sensitivity [28]. Deletion of*S6K1* induced gene expression patterns similar to those seen in CR or with pharmacological activation of adenosine monophosphate (AMP)-activated protein kinase (AMPK), a conserved regulator of the metabolic response to CR [28]. It is worthy to note, the mean life span extension was observed in female (+20.4%) but it was not increased in male *S6K1^-/-^* mice. The mean life span of oldest 10% survivors and maximum life span were also increased only in females. There was no difference in the incidence of macroscopic tumors in *S6K1^-/-^* and wild type mice. The strain differences in susceptibility to metformin could also be a reason of variability in response to treatment. It was shown that mean strain-specific lifespan varied 2- to 3-fold under *ad libitum* feeding and 6- to 10-fold under dietary restriction in virgin males and females in 41 recombinant inbred strains of mice [159]. Notably, dietary restriction shortened life span in more strains than those in which it lengthened life. Food intake and female fertility varied markedly among strains under *ad libitum* feeding, but neither predicted dietary restricted mice survival. Therefore, strains in which dietary restriction shortened life spans did not have low food intake or poor reproductive potential. These results demonstrate that life extension by dietary restriction may not be universal.

## CONCLUSION

The striking similarities have been described between insulin/IGF-1 signaling pathways in yeast, worms, flies, and mice [10]. Many characteristics of mice that are long lived due to genetic modifications resemble effects of caloric restriction in wild-type (normal) animals. Comparison of characteristics of exposed to these endogenous and exogenous influences shows a number of similarities but also some differences. Effects of antidiabetic biguanides seems to be more adequate in the prevention of age-related deteriorations in glucose metabolism and in insulin signaling pathway as well as in such important for longevity parameters as fertility and a resistance to oxidative stress and tumorigenesis than those induced by caloric restriction and genetic manipulations. It was recently demonstrated by Ligibel et al. [160] that a physical activity program significantly lowers insulin levels raises the intriguing possibility that lifestyle intervention may act as a targeted therapy in breast cancer. Metformin use in diabetic patients has been associated with reduced cancer incidence and mortality in the number of population-based studies. Furthermore, there is evidence that metformin may have insulin independent direct effects of cancer cells, acting as a mammalian target of rapamycin (mTOR inhibitor) [101,102,131-137,161-163]. It was suggested that this dual action of metformin (insulin reduction and mTOR inhibition) makes it a particularly attractive target for evaluation in breast cancer [50,51,101,102,161,164] as well as some other, e.g. colon cancer. It remains to be shown that either antidiabetic biguanides can extend life span independently of CR [3,165].
